# Maintenance of Homeostasis in the Aging Hypothalamus: The Central and Peripheral Roles of Succinate

**DOI:** 10.3389/fendo.2015.00007

**Published:** 2015-02-02

**Authors:** Thomas T. Chen, Eugene I. Maevsky, Mikhail L. Uchitel

**Affiliations:** ^1^Institute of Theoretical and Experimental Biophysics, Russian Academy of Sciences, Moscow, Russia

**Keywords:** succinate, hypothalamus, homeostasis, menopause, mitochondria, hypoxia-inducible factor-1α, mTOR, IKK-β/NF-κB

## Abstract

Aging is the phenotype resulting from accumulation of genetic, cellular, and molecular damages. Many factors have been identified as either the cause or consequence of age-related decline in functions and repair mechanisms. The hypothalamus is the source and a target of many of these factors and hormones responsible for the overall homeostasis in the body. With advanced age, the sensitivity of the hypothalamus to various feedback signals begins to decline. In recent years, several aging-related genes have been identified and their signaling pathways elucidated. These gene products include mTOR, IKK-β/NF-κB complex, and HIF-1α, an important cellular survival signal. All of these activators/modulators of the aging process have also been identified in the hypothalamus and shown to play crucial roles in nutrient sensing, metabolic regulation, energy balance, reproductive function, and stress adaptation. This illustrates the central role of the hypothalamus in aging. Inside the mitochondria, succinate is one of the most prominent intermediates of the Krebs cycle. Succinate oxidation in mitochondria provides the most powerful energy output per unit time. Extra-mitochondrial succinate triggers a host of succinate receptor (SUCN1 or GPR91)-mediated signaling pathways in many peripheral tissues including the hypothalamus. One of the actions of succinate is to stabilize the hypoxia and cellular stress conditions by inducing the transcriptional regulator HIF-1α. Through these actions, it is hypothesized that succinate has the potential to restore the gradual but significant loss in functions associated with cellular senescence and systemic aging.

## Introduction

Aging is an irreversible phenomenon in all species that is characterized by a progressive decline in all physiological functions (1–3). With improvement in public health, medical technologies, and nutrition, adults over 50 have become the fastest growing segment of society (4). However, by this age, degenerative health-related deficits start to appear and pose a potential economic burden to the individual and the society. In women, the aging is marked by the cessation of ovarian function and reproductive cycle accompanied by the decline in cardiovascular health, motor control, bone integrity, and cognitive and psychosocial faculty (5). Both sexes experience a loss of muscle tone and bone strength, a decline in energy level, a weakening of immunity, a degeneration of cognitive ability, and an increase in body fat that secondarily leads to increased risk for metabolic and cardiovascular diseases (6, 7).

Many hypotheses have been put forward to explain the cause of aging and biological bases for the functional decline, including gene-directed cell senescence, chromosome damage, DNA mis-repair, and telomere shortening [reviewed by Ref. (8–11)]. Other physiological causes are hormonal imbalance, excessive caloric intake, mitochondrial dysfunction, and oxidative stress (12–14). The landmark discovery of replication senescence by Hayflick and Moorhead (15) showing that human embryonic somatic cells could only divide a limited number of times under optimal conditions *in vitro* (15) significantly improved our understanding of aging. This phenomenon, known as Hayflick limit, is demonstrated in virtually all cell types and later shown to be due to telomere shortening after each division in cultured cells (16). Although not the only causes, cellular senescence and telomere attrition are considered two common denominators in aging (3).

Considering that aging is a multifactorial and cumulative process with multiple contributors interacting with one another in a cascade manner, it is difficult to narrow down one specific cause (17). As in many diseases, the degenerative process is a complex interplay of two main components: genetic, or preprogrammed, and phenotypic variability associated with non-genotoxic stress and environmental influences. Of the nine hallmarks summarized by Lopez-Otin, genetic instability, telomere attrition, loss of proteostasis, and stem cell exhaustion are primarily genetically predetermined. Epigenetic alterations, deregulated nutrient sensing, cellular senescence, and altered intercellular communication have their primary source in life style and environment influences. Recently, the importance of interconnection among these factors and the difference between longevity (lifespan extension) and aging (18) are also recognized. In this context, aging refers to the progressive appearance of a number of aging-induced phenotypes, i.e., reduced reproductive functions and other metabolic and adaptive changes (19).

In this review, we hypothesize that certain organs (i.e., the hypothalamus) and organelles (i.e., the mitochondrion) are more crucial than others in the aging process by virtue of the dominant role they play in the body (3, 20–22). To this end, we present a growing body of supportive evidence demonstrating that chronic low-grade inflammation of hypothalamic and other somatic cells contribute to generalized age-related degenerative processes (23). Of particular interest is the recognition that certain genes and their translational products play significant roles in the delay or progression of aging (12, 13, 24). This opens the possibility that potential therapeutic agents could be developed to target these genes and metabolites to moderate the age-associated degenerative processes and improve quality of life.

The purposes of this review are to (1) highlight the commanding role of the hypothalamus in organismal aging; (2) reassess the prominent role of the mitochondrion and the emerging role of one of its metabolites, succinate, in cellular aging; and (3) evaluate our current understanding of key genes and signaling pathways involved in the aging process. A better understanding of these important components of senescence has the potential benefit of guiding the development of effective therapeutic interventions in enhancing the quality of life of the aging population.

## The Aging Hypothalamus: Gradual Loss of Homeostatic Regulation

The hypothalamus is a collection of distinct neurosecretory cells located at the base of the brain. These neurosecretory cells receive a multitude of external and internal signals from virtually all organs in the form of hypothalamus-end-organ axes. They interpret, integrate, and respond to these messages accordingly and maintain homeostasis in the body. The vital processes under control of the hypothalamus include regulation of body temperature, nutrient intake and energy balance, sleep and wake cycle, sexual behavior, reproductive cyclicity, water and electrolyte balance, stress adaptation, nursing, growth, and circadian or ultradian cycles (6, 7, 25). When the responsiveness of these neurons declines during aging, all body activities are adversely affected. This is especially problematic for women because menopause, which generally occurs around age 50, is at the prime stage of a woman’s life history (2, 26–32). The cessation of the hypothalamus-pituitary-gonadal (HPG) function also triggers dysregulation of other homeostatic functions of the hypothalamus, i.e., loss of muscle tone and bone density (the HP-growth hormone/IGF axis), a decline in immune and adaptive responses (the HP-adrenal axis), and cognitive faculties. Of particular concern is that a woman’s menopause transition is frequently accompanied by weight gain (33), which stems from imbalance among the orexigenic [Agouti-related peptide/neuropeptide Y (NPY) neurons], anorexigenic [pro-opio-melano-cortin (POMC)/leptin neurons], and energy expenditure (orexin neurons) circuitry (the HP-adipocyte axis), disruptions in nutrient sensing (hypothalamic mTORC1 and mTORC2), and interactions with the microglial cells (34–37). Because of its impact on cardiovascular diseases and a multitude of health issues, attention has been focused on the dominant role of the hypothalamus in systemic aging. Indeed, in the large-scale Wisconsin epidemiology study, human longevity has been linked to the HPG axis (38).

Historically, one of the earliest ideas suggesting that the aging process stems from a progressive loss of hypothalamic sensitivity and homeostatic imbalance came from studies conducted in aging rodents that are corroborated with clinical conditions in humans (21). In a series of studies, Dilman and Anisimov examined changes in the thresholds of sensitivity in three major hypothalamic-pituitary-end-organ axes, namely, the reproductive, stress adaptive, and energy/thyroid systems (39, 40), II and III. In a hemi-castrated rat model, in which compensatory hypertrophy of the contralateral ovary can be investigated, the dose of exogenously administered estrogen required to suppress the compensatory effect increases as age advances from 1-month-old to 28-month-old rats. The degree of suppression is comparable regardless of whether estrogen is given systemically or directly into the third ventricle of the brain. These studies strongly suggest that the responsiveness of the hypothalamus to estrogen feedback gradually decreases with age.

To ascertain the tissue specificity of the change in sensitivity, uptake of radiolabeled estradiol by various nuclei was studied. The anterior and mediobasal hypothalamus where gonadotropin-releasing hormone (GnRH) neurons are located showed marked decreases in ^3^H-estradiol uptake with advancing age (21). Administration of l-DOPA, a D1 receptor agonist and secretagogue of GnRH (41) restores the uptake of ^3^H-estradiol by the hypothalamus, but not by the pituitary gland which serves as a control. l-DOPA also restores the ability of estrogen to suppress compensatory ovarian hypertrophy (21).

When the adaptive homeostat (adrenal axis) and the energy homeostat (growth hormone, fatty acid, and glucose metabolism) were studied using their respective feedback regulators (dexamethasone, insulin, free fatty acids, and glucose, respectively), thresholds of the hypothalamic sensitivity were raised toward these agents in both aging rodents and humans (39, 40), II and III. However, opposite effects were observed in the dopaminergic neuron-lactotroph-prolactin axis, presumably due to the fact that this axis is normally under inhibitory regulation (39, 40), IV. These observations established the concept that physiological aging stems from a progressive loss of sensitivity of the hypothalamus toward their respective feedback regulators and provided experimental evidence for the neuroendocrine theory of aging (20, 42).

Dilman and Anisimov reasoned that before menopause, the hypothalamus constantly adjusts to inputs from internal and external sources with accuracy and precision. However, with aging, the sensitivity of the hypothalamus to feedback regulators begins to decline. This results in a progressive loss of homeostasis and eventually, disruption of appropriate hormone production and an inability of the hypothalamus to appropriately regulate its target tissues. In order to maintain the same level of responsiveness, stronger feedback signals or increased sensitivity of the hypothalamus are required. This disruption of homeostasis and the age-dependent loss of responsivity are manifested with syndromes such as menopause, andropause, adrenopause, somatopause, and many other metabolic disturbances. Dilman’s hypothesis about the primary role of the hypothalamus in aging has been described in an exhaustive review of the age-related changes in the structure and function of the hypothalamus (43).

In retrospect, this neuroendocrine theory of aging is in no way in conflict with or excludes other paradigms of aging, but instead, accentuates the importance of the interconnection of many hallmarks of aging (18, 19). A modified version of this theory “the hyperfunction theory” was proposed recently that incorporates the genetic components with dysregulation of various signaling pathways during aging (13). Virtually, all the genes implicated in the hyperfunction theory, i.e., SIRT1, mTOR, NF-κB, ras, PI3K, p53, etc., play a role in uncontrolled cell division and some aspect of reproductive aging. In the following sections, advances on the three fronts that support the neuroendocrine theory of aging will be discussed. They are:

- Better understanding of the mechanism of end-organ resistance in chronic stimulation.
- Discovery of key longevity genes and their regulatory pathways.
- Determination of the role of succinate in systemic and hypothalamic metabolic adaptation.

## Better Understanding of the Mechanism of End-Organ Resistance in Chronic Stimulation

Although the neuroendocrine theory of aging is supported by experimental evidence, the exact cellular and molecular mechanisms were not known when it was first proposed. Advances in our understanding of the consequence of over-stimulation or chronic, low-grade activation of various neuroendocrine cells allow us to revisit and better interpret this theory. From a large body of research conducted since the 1980s on down-regulation and desensitization of various endocrine organs [see below], we begin to understand the underlying cellular and molecular mechanisms by which neuronal and endocrine cells terminate the action of their stimulatory signal. The accumulation of both types of over-stimulation during a life-time results in a progressive elevation of the threshold of sensitivity of the target cell toward their cognate activator. This eventually leads to the end-organ refractoriness. This phenomenon has been demonstrated in virtually all endocrine glands, including targets of insulin (44, 45), catecholamines (46, 47), corticotropin-releasing hormone (48), GnRH (49), growth hormone (50), and luteinizing hormone (LH)/hCG (51). The reduced responsiveness of the target organ is the pathological basis for diseases like insulin resistance, Type II diabetes, the “metabolic or insulin resistance syndrome” (44, 52), and hyperprolactinemia-induced infertility. The end-organ resistance is also the reason for ineffective treatment of many diseases (52–56). On the other hand, GnRH receptor-mediated desensitization of the hypothalamus is used routinely as the strategy to increase oocyte reserves for later recruitment in standard infertility treatments (49). In the stress adaptive system, it is well known that while short-term stressors promote a beneficial “hormetic stress adaptation,” prolonged exposure could shorten lifespan (57).

The cellular and molecular mechanisms for down regulation and desensitization largely depend on the cell type. The β-adrenoceptor desensitization/down-regulation is the most extensively studied (46, 47) and involves β-arrestin-mediated receptor internalization, sequestration into coated pits, and caveolae for degradation or recycling (58). In other systems, uncoupling of G-protein and its effectors, activation/inactivation of key signal transduction kinases have been demonstrated to contribute to the unresponsiveness of the target cell (44, 48, 49, 51). The age-related resistance in hypothalamic functions and reduced uptake of the feedback steroid by the hypothalamus are consistent with this model (21).

## Discovery of Key Longevity Genes and Their Regulatory Pathway

Over the last decade, several candidate genes connected to the onset and progressions of age-related degenerative process have been identified and their regulatory pathways mapped out (59–61). Some of the most extensively studied are the gene family of SIRT, TOR, NF-κB, insulin/growth factor-related genes, and some cancer-related genes such as Ras, PI3K, and p53 (13, 62). Two of the most prominent non-genetic factors regulating these genes are the accumulation of molecular damage brought about by excessive life-long caloric intake and oxidative damages from certain reactive oxygen species (ROS) (63–65). Recently, a more complete picture has emerged of the signaling network involved in dietary- and reactive oxygen-induced damage that promotes cellular senescence in mammals (19, 66). This network of molecular interaction involves three major components: (1) external signals, such as calories, energy, and hormones/growth factors; (2) an intracellular mediator, the TOR/mTOR kinases (*the mammalian target of rapamycin*) nutrient response pathways; and (3) the target gene modulator, IKK-β/NF-κB (*or the NF-κB pathway, inhibitor of nuclear factor kappa-B kinase subunit* β*/nuclear factor kappa-light-chain-enhancer of activated B cells*). The two intracellular signaling pathways, TOR and NF-κB, previously thought to be independent, are in fact, closely linked, and both are strongly influenced by dietary status and ROS (12). They are present in virtually all somatic cells in the body. But more significantly, both pathways are shown to converge into a single common signaling pathway in the hypothalamus. The discovery of these specific mediators and modulators provides a common denominator and helps to unify a number of previously independent hallmarks of aging into a model of the aging process rooted in the hypothalamus.

### mTOR and its role in the hypothalamus

mTOR is an enzyme belonging to a family of protein kinases, which are the target of the anticancer drug rapamycin (67, 68). One of the first indications that mTOR regulates cellular aging came from studies in *S. cerevisiae* (67) in which the TOR gene was deleted, resulting in doubling of the lifespan (69). Through its inhibitory action on the TOR gene family, rapamycin has been shown to extend the lifespan of diverse model organisms including worms, flies, and even mammals (65, 70). These observations firmly established that mTOR is a central, evolutionarily conserved determinant of longevity.

mTOR is activated proximally by the intake of a variety of nutrients and hormones/growth factors. After a meal, nutrients and fuels, such as glucose, activate mTOR for the anabolic synthesis of cellular carbohydrates, proteins, and lipids (65). This turns on a set of downstream effectors, including cap-dependent mRNA translation and phosphorylation of the ribosomal protein S6 kinase, leading to cell growth, accelerated metabolism that favors cell survival (68, 69, 71). Additionally, mTOR inactivates the cellular catabolic and degradation pathways and prevents cell death. The overall action of the mTOR pathway is therefore, to encourage cell growth and proliferation in the presence of nutrients under normal physiological states. However, chronic nutrition overload and overexpression of mTOR lead to oxidative stress, damage accumulation, and eventually cell senescence – all hallmarks of inflammatory responses (3). This escalation of cellular stress and the compromised ability of self-repair result in the development of age-related diseases such as cancer, obesity, type 2 diabetes, and neurodegeneration (72). Since insulin and growth factors prominently facilitate nutrient uptake by the cell, the mTOR pathway is highly sensitive to over-stimulation by insulin and other growth-promoting factors (37).

mTOR exists in two distinctly different complex forms, mTORC1 and mTORC2 (73–75). Although they exert similar effects in energy metabolism, they operate through distinct pathways. Similar activation/inactivation mechanisms for mTORC1 and mTORC2 are also demonstrated in the hypothalamus (37, 76–79). These findings are significant because of the central homeostasis role played by the hypothalamus and the fact that neurosecretory cells are not renewed (2). In the hypothalamus, mTORC1 centrally regulates food intake and body weight through leptin and ghrelin signals and peripherally controls adipogenesis, lipogenesis, and gluconeogenesis in tissue such as the liver (73). mTORC2, on the other hand, regulates neuronal cell number, size, morphology, synaptic connectivity, and thus, plays a crucial role in the central nervous system (CNS) regulation of energy balance. In a series of elegant experiments, it is shown that chronic exposures to even low-grade nutrients such as glucose and insulin/growth factors could directly or indirectly (through the downstream regulatory enzyme *Akt*, which is also an activator of mTORC1) down regulate mTOR activity in the hypothalamus. This eventually leads to the loss of sensitivity of various hypothalamic nuclei and their ability to regulate their respective homeostatic loops (37, 75–79).

### The IKK-β/NF-κB complex in the hypothalamus

A major downstream target of mTOR has recently been identified (12, 80). This target is a fast-acting cell survival protein complex and transcription factor known as the IKK-β/NF-κB complex (77–79). NF-κB can influence the expression of hundreds of genes involved in cellular inflammatory responses and has been studied as one of four marker genes in people with exceptional longevity (81). Under normal conditions, NF-κB exists in an inactive form. Environmental pro-inflammatory agents, including caloric excess and oxidative stress can trigger the activation of the IKK-β/NF-κB pathway. The same regulatory pathway exists in the hypothalamus as well. Recently, Tang and Cai showed that chronic caloric excess leads to inflammatory responses in the mediobasal hypothalamus, precisely the same area affected in the study of Ref. (21). This inflammation is shown to involve an IKK-β/NF-κB-dependent pathway in microglial cells, a modified macrophage. Microglial cells are in constant communication with hypothalamic neurosecretory cells through pro-inflammatory cytokines, TNFα and IL-1β (79, 82), thus activating the IKK-β/NF-κB system in these cells. Over time, this leads to signal resistance and loss of hypothalamic homeostatic responsiveness (12, 21, 65, 79). This central dysregulation has been associated with systemic aging and the accelerated development of aging-related metabolic syndromes, obesity, Type II diabetes, cardiovascular diseases, cognitive degeneration, and reproductive dysfunctions (37, 83). In the same study (37), activation of the IKK-β/NF-κB pathway is shown to strongly inhibit GnRH gene transcription (79). This gives additional credence to the central role of the hypothalamus in systemic aging and age-related degeneration of body functions.

### Hypoxia-inducible factor 1α – key transcription factor in nutrient sensing in the hypothalamus

The role of the hypothalamus as the center that controls appetite and nutrient intake is well established. The hypothalamus achieves this regulation in two ways: through positive and negative feedback by numerous hormones including leptin and insulin (83–86), as well as nutrient sensing by molecules such as glucose, intermediary metabolites, amino acids, and fatty acids (74, 87, 88). Hormone-sensing is thought to provide a long and sustained homeostatic regulation of body weight, while nutrient-sensing offers a short-term regulation of energy balance.

In studying the downstream modulators of the mTOR pathway in the hypothalamus, a nuclear transcription factor, HIF-1α is identified to play a key role in nutrient-sensing. From its namesake, HIF-1α is one of the first responders during cellular and systemic hypoxia that help an organism to deal with oxygen deficiency (89). It achieves this task in multiple ways. HIF-1α activates the anaerobic breakdown of sugar to provide urgently needed energy for cell survival under low oxygen conditions (the glycolysis pathway). It stimulates more red blood cell formation (erythropoiesis) by the bone marrow (90, 91) and new blood vessel formation (angiogenesis) in (hypoxia) affected areas (92). The combined action helps increase energy flow, oxygen-carrying capacity, and nutrient delivery, and thus, ensures the survival of the organism.

The level of HIF-1α is stringently regulated in response to the level of oxygen in the microenvironment (76). In the presence of an adequate amount of oxygen, HIF-1α is targeted for degradation because it is not needed. But under hypoxic conditions, the synthesis of HIF-1α is activated and the degradation is inhibited, resulting in more HIF-1α to help restore oxygen homeostasis. The combined action ensures the availability of sufficient oxygen and energy for cell survival.

HIF-1α is recently found to play a key role in glucose sensing and the metabolism of other intermediary metabolites in the hypothalamus. The particular area of the hypothalamus identified as the site of glucose sensing and energy regulation coincides with an area rich in POMC-expressing neurosecretory cells (93–95). The POMC gene has long been recognized to assist in the acute and long-term adaptation of an organism to various types of stress. The POMC gene product, pro-opio-melano-cortin, is the precursor protein that gives rise to many potent trophic hormones in the “master gland,” the pituitary. Some of the hormones produced by the pituitary gland are melanocyte-stimulating hormones (MSHs), corticotrophin (ACTH), and β-endorphin. MSHs and ACTH are collectively known as melanocortins and are the dominant regulator of feeding behavior.

Because of the blood-brain barrier, glucose is the primary source of energy in the mammalian brain (96). The increase in glucose concentration is sensed by the nutrient sensor of the hypothalamic POMC neurons. Working through the mTOR nutrient-sensing pathway, the synthesis of HIF-1α is stimulated and its degradation suppressed, resulting in an enhanced HIF-1α (97). One major action of the HIF-1α is to turn on the POMC gene and thus, increase melanocortins. The HIF-1α and POMC pathway is therefore an important circuit in the hypothalamic control of appetite and energy balance. As the hypothalamus ages, the sensitivity to the sensor input begins to decline which results in dysregulation of feeding behavior (93, 94, 97). Internal or external signals that activate this pathway will thus be useful therapeutic targets in preventing weight gain, obesity, and other metabolic or cardiovascular diseases (97–99).

## The Role of Succinate and Other Metabolic Intermediates in Metabolic Adaptation

Among many nutrient sensors and regulators in the hypothalamus, there is strong evidence indicating the Krebs cycle intermediates, succinate and fumarate, play an obligatory role in restoring energy homeostasis (100–104). Succinate is one of the most important raw materials in the energy-producing cycle that generates adenosine triphosphate (ATP) and fumarate is produced from succinate by the oxygen-dependent succinate dehydrogenase (101).

**Figure 1:**
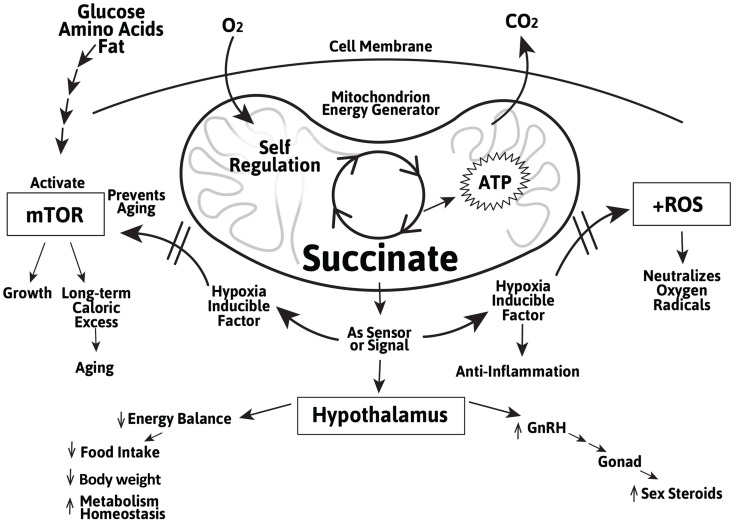
**Schematic representation of the role of the Krebs cycle intermediate succinate as a Krebs cycle intermediate in the mitochondrion and as an extra-mitochondrial sensor/signal in the regulation of cellular growth and aging mediated by mTOR and induction of the cellular stress regulator HIF-1α and inhibiting of the ROS in peripheral tissues and in the hypothalamus**. The actions of succinate in the hypothalamus include energy balance, metabolic homeostasis, and GnRH gene expression.

In addition to their ATP and nicotinamide adenosine dinucleotide (NADH)-generating role in energy metabolism, succinate, and to a lesser extent, fumarate, are involved in sensing and regulating the metabolic activity during cellular stress, including during hypoxia and exercise. This allows most organisms to coordinate and adapt to transient or prolonged oxygen-deficit conditions. When oxygen is abundant, the Krebs cycle generates ATP and NADH which are used for virtually all cellular activities. When oxygen is insufficient, the Krebs cycle runs in reverse resulting in an accumulation of succinate in the mitochondrial matrix (105). This has been demonstrated in activated macrophages and is thought to be critical for the body’s immune defense. An accumulation of succinate and fumarate in the cytosol directly induces the synthesis and stabilization of the transcription factor HIF-1α independent of the mTOR pathway by inhibiting proteolysis of HIF-1α by the prolyl hydroxylase domain (PHD)-containing enzyme (82, 106). In the hypothalamus, succinate also importantly stabilizes HIF-1α and promotes POMC gene expression for the central control of food intake and energy expenditure (76, 107). Thus, succinate not only helps restore energy production through glycolysis under hypoxic conditions, it also helps regulate the complex leptin-mediated behavior in the appetite center and long-term weight control (108–112). These findings affirmed and corroborated with the decade-long work of Maevsky, Kondrashova, and their colleagues that succinate (compared to fumarate and other metabolites) is an effective modulator in the central regulation of energy expenditure, weight control, and metabolic homeostasis (113). It suggests a potential role of succinate in the regulation of a multitude of hypothalamic function in adults and particularly during the aging process (Figure 1).

It should be noted that succinate is not the only intermediate metabolite studied and demonstrated to have beneficial effects in receptor-mediated adaptive actions. Receptors for other Krebs cycle intermediates such as α-ketoglutarate have also been identified (114, 115). Both fumarate and succinate mediate the glucose-induced up-regulation of HIF-2α (76). Malate and fumarate have been shown to extend lifespan in *C. elegans* (116). Although succinate does not extend lifespan *per se* in *C. elegans*, it does activate the longevity regulator DAF-16 and its nuclear translocation as malate and fumarate do, and all three increase stress resistance (76). Succinate is also shown to be a biomarker of ischemia and can also be formed from several sources, i.e., the glyoxylate pathway, glutamine, aspartate, and gamma amino butyric acid (GABA) metabolism (117). Succinate at a dose of 2 mg/kg of body weight results in increased blood flow, synthesis, and release of catecholamines, which in turn, stimulate the hypothalamic centers to enhance local blood flow (118). Taken together, succinate may play a more dominant role than previously thought in age-related metabolic adaptations.

## Identification of the Extra-Mitochondrial Role of Succinate and Surface Succinate Receptors in Peripheral Tissue

While succinate is well known for its role as an intermediary metabolic product in the Krebs cycle, accumulation of succinate in mitochondria due to hypoxia may cause it to diffuse out into the general circulation through a number of putative membrane transporters (105) and may act as a signaling molecule in peripheral tissues. Succinate was shown to mediate the action of adrenalin and other neurotransmitters (118–120). This suggests a non-cell autonomous role of succinate that diverges from its traditional role as simply a raw material in the production of ATP in the mitochondria.

A cell surface receptor that specifically binds succinate was subsequently identified in 2004 (114) and its signal transduction pathway was later elucidated (121–125). Many of the extra-mitochondrial actions of succinate have since been substantiated by studies in many tissues (see below). The receptor, GPR91 or SUCNR1, is a G-protein-coupled receptor that is closely related to the family of P2Y purinoceptors (105, 114). The mRNA of SUCNR1 was found to be highly expressed in the kidney and to a lesser extent, in the liver and the spleen (114, 121, 126–128). Later it was localized in many tissues including cardiomyocytes, bone marrow hematopoietic precursor cells, immune cells (129, 130), the retina, and adipocytes (121–123). Many of these are part of the hypothalamic-pituitary-end-organ homeostatic axes. More recently, succinate has been shown to play an important role in nutrient sensing, energy balance, stress adaptive, and GnRH regulation in the interaction between microglial cells and the hypothalamus (37, 76).

The SUCNR1 is coupled to at least two signaling pathways – Gi/o and Gq, depending on the tissue studied (114, 130, 131). Interaction of succinate with SUCNR1 results in increases of calcium and inositol phosphate, and decreases in cyclic adenosine monophosphate (cAMP) formation. On the other hand, succinate stimulates cAMP production in myocardiocytes and platelets as well as calcium accumulation (122). Similar to other GPCRs, SUCNR1-ligand interaction results in internalization of the receptor, desensitization, and subsequent sequestration and receptor recycling (114, 132). The estimated half-maximal effective concentration of succinate is compatible with the physiological concentration found in the body fluid (123). All these lines of evidence point to the fact that succinate plays important regulatory roles in immune response, lipid metabolism, formation of blood cells and blood vessels, and restoring blood pressure and cardiovascular function. Because of its pivotal role in the regulation of the central and peripheral organs, considerable interests have been raised in the development of agonists and antagonists to modify various vital functions. One peripheral role of succinate that is of concern is its hypertensive action mediated by renin secreted by the macula densa of the juxtaglomerular apparatus of the kidney as a result of SUCNR1 activation. This is demonstrated in isolated tissue preparations and in spontaneously hypertensive rats (SHR) (133, 134). However, whether or not the same hypertensive effect can be reproduced in humans requires additional studies as many of the effects observed in isolated tissue preparation and in rodents have not been demonstrated in humans (117, 135).

Of all the actions the succinate receptor mediates in human physiology and pathology, the most intriguing one is that SUCNR1 serves in homeostasis as a sensor for extracellular succinate. That is, under normal energy balance, mitochondrial succinate serves its principal role in energy production in the Krebs cycle. Neither accumulation of succinate in the mitochondria nor leakage of succinate out of the mitochondria occurs, and the peripheral SUCNR1 remains inactive (105). Under stressful conditions, as in hypoxia, hyperglycemia (as in diabetes), hypertension, or liver insult, succinate accumulates in mitochondria and subsequently diffuses out and into the circulatory system (136), in Ref. (105). Extracellular succinate, in turn, binds to SUCNR1 in various tissues and promotes tissue-specific action (105, 115, 133, 134). More recently, it is shown that succinate (and its derivative fumarate) induces the synthesis of anti-inflammatory proteins, stress-adapting hormones, and GnRH gene expression, and also suppresses feeding behavior – all homeostatic circuitries regulated by the hypothalamus (discussed above and summarized in Figure 1). These findings provide molecular evidence of the neuroendocrine theory of aging proposed over 40 years ago. Deeper understanding of these molecular pathways may point to finding solutions to restoring homeostasis of the body. Given that aging is associated with multiple organ degeneration, it would be of limited benefit to treat individual organs and their diseases. The hypothalamus, a regulator of multiple physiological tissues and processes, offers a single target for potential drug therapy.

## A Potential Therapeutic Role of Succinate in Metabolic Diseases and Menopausal-Related Symptoms

Two pressing issues in moderating the degenerative process in human aging are weight control (which leads to cardiovascular diseases, metabolic syndromes, and cancer) and menopausal symptom relief. Intriguing questions are raised about the potential benefit of using succinate in alleviating age-associated metabolic disturbances. In a series of studies, succinate is shown to facilitate the formation of glutamate, which increases the turnover of adenylate and glutamine and induces nitric oxide synthesis in the brain. This results in stimulation of the vascular tone, blood flow, antioxidant activity, and improvement of age-related degenerative changes (137).

Another succinate target in the hypothalamus is the hypothalamus-POMC axis involved in nutrient sensing as well as other functions including the regulation of sexual behavior, lactation, the reproductive cycle, and possibly central neural control (97). This strongly suggests that regulation of HIF-1α activity by succinate as shown in previous sections may have multiple benefits.

A pulsatile GnRH secretion pattern is essential for follicle-stimulating hormone (FSH)/LH release by the pituitary gland and the integrity of positive and negative feedback loops involving estrogens (7). An added evidence of the importance of the homeostatic feedback between the gonad and the hypothalamus is the illustration that mitochondria are a major target of estrogen (138) and that estrogen regulates mitochondrial metabolism in the hypothalamus (139).

From recent groundbreaking discoveries of mTOR and NF-κB pathways in the aging hypothalamus, one of the casualties of an overactive NF-κB is the suppression of GnRH gene expression, and a reduction in GnRH synthesis and secretion (37). Since GnRH is the key regulator of FSH and LH and secondarily controls the gonadal production of estrogen and testosterone (7), many reproductive and non-reproductive targets controlled by these sex steroids are adversely affected. These include the cessation of the menstrual cycle with its associated menopausal symptoms, a loss of muscle tone and bone strength, a decline in the energy level, a weakening of the body’s ability to defend against infectious agents, a degeneration of cognitive ability, and an increase in body fat that could lead to heart diseases and cancer (140, 141). Administration of GnRH or rapamycin (an mTOR inhibitor) in animals improves many age-related symptoms, including skin atrophy, muscle weakness, and bone loss (37). Therefore, in addition to the reproductive role, GnRH is shown to act on the brain and peripheral organ systems to regulate systemic aging, albeit through a different signaling pathway from rapamycin (37, 107). The finding that hypothalamic inflammation is responsible for the shutdown of GnRH and systemic aging provides an additional molecular mechanism supporting the neuroendocrine basis for aging. This may also lead to the development of treatments to slow down the course of aging and relieve age-related symptoms. Although GnRH and rapamycin are shown to reverse the aging process, both have undesirable side effects. Succinate as a natural molecule that directly activates the HIF-1α/POMC pathway may prove to be an ideal candidate in this regard. Indeed, POMC neurons are shown to project and make synaptic contacts with GnRH neurons and POMC-derived neuropeptides elicit a robust activation of the GnRH/LH axis in different mammalian species (34, 142).

## Conclusion

Aging is a multi-faceted decline of body functions. Many hypotheses have been proposed to explain the cause of aging. They include permanent damage of the genetic material, hormonal imbalance, and environmental/lifestyle insults, such as excessive caloric intake, free radical or oxidative stress, and inflammation.

The hypothalamus is the master regulator of homeostasis in vertebrates and is the source and target of continual regulatory adjustments throughout one’s lifetime. The neuroendocrine theory of aging proposed by Dilman over 40 years ago postulates the functional decline of the hypothalamus is due to a decrease in its sensitivity toward feedback control.

Succinate is not only an important intermediary metabolite in the energy generating Krebs cycle, but also has diverse extra-mitochondrial roles, acting in both cell and non-cell autonomous manner in peripheral tissues. The concept of succinate as a signaling molecule is confirmed by the identification of the succinate receptor, elucidation of its signal transduction pathways, and recognition of its diverse action in many tissues.

Recently, mTOR has been identified as a proximal molecular switch in the onset and progression of systemic aging. Over-stimulation of hypothalamic mTOR as a result of chronic exposure to nutrients and activation of the pro-inflammatory NF-κB that inhibits expression of the GnRH gene are responsible for the loss of sensitivity of the hypothalamus. Activation of HIF-1α which activates POMC gene expression appears to reverse the decline of hypothalamic function.

Succinate stimulates the expression of the HIF-1α gene, stabilizes the HIF-1α protein, and mitigates the functional decline in both the stress adaptation/energy-balancing pathway and the GnRH loop. This helps to moderate age-related processes that lead to weight gain, menopausal symptoms, and other degenerative diseases. Since the succinate receptor is widely distributed throughout the body, succinate may play a central role in reversing the gradual but significant dysregulation of the hypothalamus as well as peripheral cellular senescence.

## Author Contributions

The authors have made the following declarations about their contributions: conceived and designed studies cited in this manuscript: EM and MU. Drafting and revising the manuscript: EM, MU, and TC. Final approval of the work: EM, MU, and TC.

## Conflict of Interest Statement

Eugene Ilich Maevsky, Mikhail Lvovich Uchitel, and Thomas T. Chen serve as scientific advisers to Lunada Biomedical.

## Abbreviations

ATP, adenosine triphosphate; cAMP, cyclic adenosine monophosphate; CNS, central nervous system; FSH, follicle stimulating hormone; GABA, gamma amino butyric acid; GnRH, gonadotropin-releasing hormone; LH, luteinizing hormone; NADH, nicotinamide adenosine dinucleotide; NPY, neuropeptide Y; PHD, prolyl hydroxylase domain; POMC, pro-opio-melano-cortin.
